# A Method by Which to Assess the Scalability of Field-Based Fitness Tests of Cardiorespiratory Fitness Among Schoolchildren

**DOI:** 10.1007/s40279-016-0553-6

**Published:** 2016-05-26

**Authors:** Sarah Domone, Steven Mann, Gavin Sandercock, Matthew Wade, Chris Beedie

**Affiliations:** 1 ukactive Research Institute, 26-28 Bedford Row, London, WC1R 4HE UK; 2 Centre for Sport and Exercise Science, School of Biological Sciences, University of Essex, Colchester, UK; 3 School of Human and Life Sciences, Canterbury Christ Church University, Canterbury, Kent, UK

## Abstract

Previous research has reported the validity and reliability of a range of field-based tests of children’s cardiorespiratory fitness. These two criteria are critical in ensuring the integrity and credibility of data derived through such tests. However, the criterion of scalability has received little attention. Scalability determines the degree to which tests developed on small samples in controlled settings might demonstrate real-world value, and is of increasing interest to policymakers and practitioners. The present paper proposes a method by which the scalability of cardiorespiratory field-based tests suitable for school-aged children might be assessed. We developed an algorithm to estimate scalability based on a six-component model; delivery, evidence of operating at scale, effectiveness, costs, resource requirements and practical implementation. We tested the algorithm on data derived through a systematic review of research that has used relevant fitness tests. A total of 229 studies that had used field based cardiorespiratory fitness tests to measure children’s fitness were identified. Initial analyses indicated that the 5-min run test did not meet accepted criteria for reliability, whilst the 6-min walk test likewise failed to meet the criteria for validity. Of the remainder, a total of 28 studies met the inclusion criteria, 22 reporting the 20-m shuttle-run and seven the 1-mile walk/run. Using the scalability algorithm we demonstrate that the 20-m shuttle run test is substantially more scalable than the 1-mile walk/run test, with tests scoring 34/48 and 25/48, respectively. A comprehensive analysis of scalability was prohibited by the widespread non-reporting of data, for example, those relating to cost-effectiveness. Of all sufficiently valid and reliable candidate tests identified, using our algorithm the 20-m shuttle run test was identified as the most scalable. We hope that the algorithm will prove useful in the examination of scalability in either new data relating to existing tests or in data pertaining to new tests.

## Key Points

**Table:** 

Previous research has reported the validity and reliability of a number of tests of children’s fitness.
Our systematic review indicated that the 5-min run test did not meet accepted criteria for reliability, whilst the 6-min walk test failed to meet the criteria for validity.
We further identified that of all sufficiently valid and reliable tests of children’s fitness, the 20-m shuttle run test was identified as the most scalable.

## Introduction

The health and fitness of children is increasingly recognised as a core component of public health. Two reasons for this growing emphasis are evident. Firstly, poor health adversely affects the quality of life, and the physical, academic and social development of children. Second, poor health in childhood may predispose to certain diseases and is often therefore predictive of poor health in adulthood [1]. To this end, the UK Chief Medical Officer [2] stated “the introduction of a standardised school-based fitness assessment in England may have multiple benefits that extend beyond the benefits for the individual”. Such assessment could focus on the measurement of physical activity, and/or the measurement of the results of physical activity. Methods might range from the very basic such as the total time children spend in physical education (PE) lessons and/or the number of children who take part in extracurricular physical activity, to the more complex, such as the evaluation of motor skills and physical literacy, and/or the measurement of cardio-respiratory fitness.

However, none of the above measures are currently mandated in UK schools. The current mandated measure, the National Child Weight Measurement Programme (NCMP, http://www.hscic.gov.uk/ncmp), measures body mass index (BMI). The NCMP represents one of, if not the only, proxy measures of a child’s health across the UK. Given its broad coverage, it provides valuable data on child health at a local and national population level. Arguably, however, the BMI of a child is a crude metric at best, often saying as much about genetics and somatotype as about physical activity levels and health. In fact BMI in young childhood is at best only moderately predictive of subsequent adult health status [3].

Public health agencies in the UK are encouraging novel interventions to increase levels of childhood physical activity. However, the widespread lack of routine data collection identified above renders it problematic to evaluate the true impact of any such interventions. It also renders it almost impossible to set benchmarks, to identify local pockets of excellence (or indeed underperformance), or to calculate the cost-effectiveness of interventions. Whilst many areas of public health policy are characterised by a clear evidence-based strategy, decisions relating to the health and fitness of the nation’s children are often made in an evidence vacuum.

In the short- to medium-term what is required is a means of testing the health and fitness of children that is not only valid and reliable, but is also ethical and cost-effective. It is also abundantly clear that any large-scale fitness testing of children would need to be conducted in the field as opposed to the laboratory, as the provision of resources required for the latter would be prohibitive in the extreme.

The decision as to which test should be used is challenging. Data pertaining to the reliability and validity of tests of children’s fitness are widely available. For example, Castro-Piñero et al. [4] conducted a systematic review of the criterion related validity of field based fitness testing methods in children. The results of 73 studies suggested strong support for the 20-m shuttle run test as a valid means by which to estimate cardiorespiratory fitness in children and adolescents. Likewise, Artero et al. [5] conducted a systematic review to determine the reliability of children’s fitness testing methods and reported the most reliable field-based test of cardio-respiratory fitness was the 20-m shuttle run test.

However, whilst validity and reliability are of critical importance, in the field-test context it is often required that further criteria are met. Whilst receiving little attention in the scientific literature, the criterion of scalability, that is the potential for the extension into real-world policy and/or practice of interventions or tests shown to be efficacious in controlled settings [6] is often critical to policymakers and practitioners.

### Aims of the Present Review

Our aim is to propose a novel framework by which researchers and practitioners might assess the scalability of field-based fitness tests appropriate for primary school children aged 8–11 years. We propose an algorithm by which the scalability of a candidate test can be evaluated. We then apply this algorithm to data identified via a systematic review to assess the scalability of children’s fitness tests.

## Methodology

### Identification of Components of Scalability

Scalability is to all intents a latent variable and cannot be directly measured. In order to overcome this, a collection of items or components hypothesised to co-vary with the latent variable were identified used as a proxy measurement [7].

Whilst the concept of scalability is becoming progressively more significant in public health, there is only limited information relating to its definition and core constituents. Terms used to described scalability have been applied in many different ways and contexts, with little consistency or rigour [6]. In an attempt to bring some clarity to terminology used, Milat et al. [6, 8] proposed eight core constituents: (1) delivery, (2) effectiveness, (3) cost-effectiveness, (4) evaluation, (5) reach and adoption, (6) evidence of operating at scale, (7) resource requirement and (8) practical implementation issues.

We adapted the eight criteria proposed by Milat et al. [6] to six components for the specific case of field-based fitness testing methods. Some components were represented by a single variable, whilst other components were constructed using multiple variables. These components and related variables are presented in Table 1.

**Table 1:** Scoring schedule for components of scalability

	Variable	Operational definition	Assessment criteria	Maximum score	Weight
Delivery	Test context	Can this test be conducted in a school setting?	(2) Strong evidence (1) Moderate evidence (0) Limited or no evidence	2	
	Test duration	Can this test be carried out within the time limits of a normal PE lesson?	(2) Strong evidence (1) Moderate evidence (0) Limited evidence or no evidence, i.e. test duration is longer than a normal PE lesson	2	
	Testing interval	Is this test suitable for use within a longitudinal testing programme?	(2) Strong evidence (1) Moderate evidence (0) Limited or no evidence	2	
	Delivery staff	Can the test be administered by PE teachers and/or school staff?	(2) Strong evidence (1) Moderate evidence (0) Limited or no evidence, i.e. test must be administered by researchers or clinicians with specialist skills	2	
Total				8	1
Evidence of operating at scale	Sample size	Is this test appropriate for population level testing?	(2) Strong evidence = field test administered at a national or international level (1) Moderate evidence = field test has been implemented in multiple testing settings within a local area (0) Limited or no evidence = small sample used/singular school	2	
	Number of schools	Is implementation of this field test likely to be acceptable to multiple target schools when scaled up?	(2) Strong evidence (1) Moderate evidence (0) Limited or no evidence	2	
Total				4	2
Effectiveness	Validity	Is the criterion-related validity of the test acceptable for the target population?	(2) Strong evidence (1) Moderate evidence (0) Limited or no evidence	2	
	Test–retest reliability	Is the test–retest reliability validity of test acceptable for the target population?	(2) Strong evidence (1) Moderate evidence (0) Limited or no evidence	2	
	Reach and adoption	Is there a high level of participation of the intended target population?	(2) Strong evidence (1) Moderate evidence (0) Limited or no evidence	2	
	Completion rates	Can the test be completed safely and is the test acceptable to the target participants?	(2) Strong evidence (1) Moderate evidence (0) Limited or no evidence	2	
Total				8	1
Cost	Cost effectiveness	Is the test affordable?	(2) Strong evidence = i.e. NCMP estimated cost is £123,000 based on collection of annual data from 147 PCTs (3 person days each) (1) Moderate evidence (0) Limited (i.e. not affordable) or no evidence	2	
Total				2	4
Resource requirements		Are there additional requirements in terms of equipment, space, skills, competencies and workforce requirements?	(2) None (1) Some investment required to run test (0) Resource requirements unsustainable or no evidence	2	
Total				2	4
Practical implementation issues		Can the field test be undertaken, administered and scored with ease?	(2) Strong evidence (1) Moderate evidence (0) Limited or no evidence, i.e. practical implementation issues make this test unfeasible to administer	2	
Total				2	4

*PE* physical education, *NCMP* National Child Measurement Programme, *PCT* primary care trust

### Algorithm Construction and Scoring

We constructed an algorithm as the sum of weighted scores for each of the core constituents of the scalability framework:

$$ \left\{ {x_{1} , x_{2} , x_{3} , \ldots, x_{n} } \right\} $$

In this algorithm each *x*_*n*_ represented one core constituent n of the scalability framework, i.e. delivery. Constituents of the algorithm were weighted as described below:

$$ X_{\text{score}} = \mathop \sum \limits_{i = 1}^{n} w_{i} x_{i} $$

Each single variable of a component could take a value from 0 to 2, and these variable scores were summed to produce each component score. A maximum of 8 points was possible for each of the six components (Table 1), resulting in a possible maximum scalability score of 48 for each test. We had no a priori reason to justify weighting certain components more heavily than others, so by increasing the weighting of components with low numbers of variables we were able to ensure that each component contributed equally to the overall score (however, excluding tests that did not meet validity and reliability criteria in effect weighted these two variables highly in the scalability analysis).

### Systematic Review

To facilitate the testing of the scalability algorithm, a systematic review of studies reporting tests of children’s fitness was conducted. The objective of this review was to ensure that we only established the scalability of tests that demonstrate sufficient validity and reliability.

#### Inclusion Criteria

To be included in the review, papers had to report a study of one or more of the fitness tests addressed in two recent systematic reviews [4, 5], namely the 20-m shuttle run, 1-mile run, 6-min walk, and 5-min run. Fitness tests meeting these criteria were assessed against three criteria likely critical to the successful implementation of fitness testing of schoolchildren; the validity of the test for use with children aged 8–18 years old, the reliability of the test in this age group, and the applicability of the test, that is whether a test could be implemented in a school setting as part of usual PE lessons, albeit by specially trained staff. These primary criteria were considered fundamental to the child fitness measurement scenario described in the introduction.

#### Evidence Criteria

**Fig. 1:**
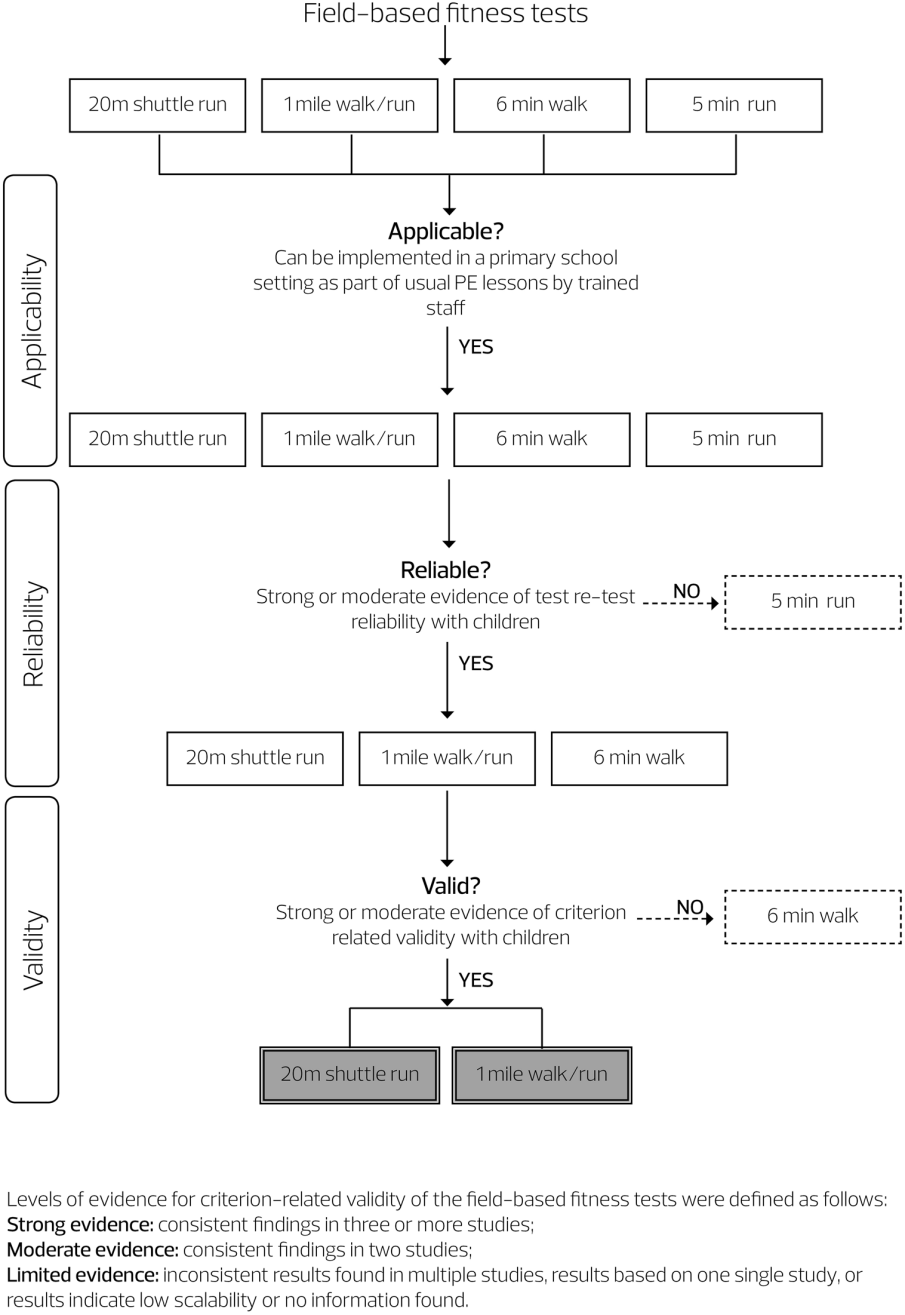
Flowchart of test assessment.* PE* physical education

A three-tier classification of evidence quality was used [4], albeit in this case referring to the validity and reliability of the tests: (1) strong evidence, that is consistent findings in three or more studies; (2) moderate evidence, that is consistent findings in two studies; and (3) inconsistent results found in multiple studies, results based on one single study, or results indicate low scalability or no information found (Fig. 1).

#### Literature Search

The literature search was undertaken between May and July 2015 using the PubMed database. Key words searched, using multiple combinations of AND/OR phrases, included ‘cardiorespiratory fitness’, ‘children’, ‘testing’, ‘field’, ‘youth’, ‘adolescents’, ‘CRF’, as well as individual test names. Further papers were identified via examining reference lists of publications already identified.

#### Data Extraction

Operational definitions for scalability characteristics are presented in Table 2. Studies were assessed on whether data relating to these characteristics were reported (Table 3). Information relating to delivery, effectiveness, cost-considerations, resource requirement and practical implementation issues were all extracted. A further data extraction form was created to capture information in studies that had directly assessed some aspect of scalability. Items in this form included ease of integration into usual service delivery, burden on delivery staff, preparation requirements, test duration, reach and adoption, completion rates, resource requirements, practical implementation issues and considerations. These are presented in Table 4.

**Table 2:** Details of review items relating to scalability framework

Component	Variable	Operational definition	Assessment criteria
Delivery	Test environment	Information relating to whether the field testing was conducted in a school setting	Yes = test performed in a school setting No = test not performed in a school setting NR
	Test duration	Expected or actual duration of the field test protocol reported	Yes = duration of test/trial reported NR
	Testing interval	Duration relating to the interval over which the testing was conducted	Yes = duration reported NR
	Delivery staff	Information relating to the personnel used to administer the testing protocols and record the results	Yes = tests performed by usual service delivery staff (PE teachers) No = Researchers or clinicians administered tests NR
Evidence of operating at scale	Sample size	Evidence that the field test has been used to assess fitness of young people at a national/population level	Yes = field test administered at a national or international level Partial = field test has been implemented in multiple testing settings within a local area No = small sample used/single school
	Number of schools	Evidence that the implementation of the field test is likely to be acceptable to multiple target schools when scaled up	Yes = multiple schools used in study No = single or no school used NR
Effectiveness	Validity	How well a specific test measures what it intends to measure	Yes = strong or moderate evidence of acceptable criterion related validity of test No = limited evidence
	Test–retest reliability	The consistency of performer/s scoring over repeated rounds of testing	Yes = strong or moderate evidence of acceptable test–retest reliability No = limited evidence
	Reach and adoption	Differential effect, reach and adoption across target groups, socioeconomic status and settings	Yes = reach and adoption is reported NR
	Completion rates	Measure of acceptability to individuals	Yes = completion rates are reported NR
Cost considerations	Cost effectiveness	Information relating to the cost of the field test per head is provided	Yes = cost per head of test is reported NR
	Resource requirements	Information relating to the required resources in terms of equipment, space, skills, competencies, workforce, and financial requirements provided	Yes = resource requirements are reported Partial = only limited reporting concerning some elements NR
	Practical implementation issues/considerations	The ease with which the field test can be undertaken, administered and scored	Yes = feasibility/practicality is discussed Partial = only limited reference to practicality issues included in discussion NR

*PE* physical education, *NR* not reported

**Table 3:** Scalability properties of reviewed articles

Field test	Study	Sample size (*n*)	Match to review criteria	Schools (*n*)	Delivery	Test duration	Testing interval	Staff	Reach and adoption	Completion rate (%)	Cost-effectiveness	Resources required	Practical issues
20-m shuttle run													
	Baquet et al. [9]	503	No	NR	Yes	NR	70 days	Yes	No	100 %	NR	Partial	Partial
	Beets and Pitetti [10]	241	No	No = 1	Yes	NR	21 days	Yes	Yes	100 %	NR	NR	Yes
	Boddy et al. [11]	27,942	Partial	Yes >1	No	NR	12 years	No	Yes	NR	NR	NR	Partial
	Borehamet al. [12]	1015	Yes	Yes = 16	NR	NR	NR	No	Yes	78 %	NR	Partial	NR
	Burns et al. [13]	134	Partial	Yes = 3	Yes	NR	NR	No	Yes	NR	NR	Partial	NR
	Castro-Piñeiro et al. [14]	2752	Partial	Yes = 18	NR	NR	NR	NR	Yes	95 %	NR	Yes	NR
	Jenner et al. [15]	1311	Partial	Yes = 27	Yes	NR	4 months	No	Yes	84 %	NR	Partial	NR
	Kim et al. [16]	6297	Partial	Yes = 15	Yes	NR	3 years	Yes	Yes	89 %	NR	Partial	NR
	Mahar et al. [17]	266	Partial	Yes = 26	Yes	NR	7 days	Yes	No	NR	NR	Partial	NR
	Mahoney et al. [18]	103	No	No = 1	Yes	NR	28 days	No	Yes	100 %	NR	Partial	Partial
	Matsuzaka et al. [19]	132	Partial	Yes = >1	NR	NR	2 months	NR	Yes	NR	NR	Partial	NR
	Ortega et al. [20]	123	Yes	No = 0	No	NR	14 days	No	Yes	100 %	NR	Partial	NR
	Ortega et al. [21]	3528	Yes	No = 0	No	90 mins^a^	2 years	No	Yes	NR	NR	Partial	NR
	Quinart et al. [22]	30	No	No = 0	No	NR	9 months	No	Yes	88 %	NR	NR	Partial
	Roberts et al. [23]	15,315	Partial	Yes = >1	Yes	NR	4 years	No	Yes	NR	NR	NR	NR
	Sandercock et al. [24]	2041	Partial	Yes = 5	Yes	NR	3 months	No	Yes	NR	NR	Yes	NR
	Sandercock et al. [25]	6628	Partial	Yes = 28	Yes	NR	1 years	No	Yes	NR	NR	Partial	NR
	Sandercock et al. [26]	7393	Partial	Yes = 26	Yes	NR	4 years	No	Yes	NR	NR	Partial	NR
	Stratton et al. [27]	15,621	Partial	Yes = 106	NR	NR	6 years	No	Yes	74 %	NR	NR	NR
	Voss and Sandercock [28]	208	No	NR	Yes	NR	3 months	No	No	NR	NR	Yes	NR
	Voss and Sandercock [29]	5927	Partial	Yes = 23	Yes	NR	1 years	No	Yes	NR	NR	Partial	NR
	Voss and Sandercock [30]	4029	Partial	Yes = 26	Yes	NR	1 years	No	Yes	NR	NR	Partial	NR
1-mile walk/run													
	Beets and Pitetti [10]	241	No	No −1	Yes	NR	21 days	Yes	Yes	100 %	NR	NR	Yes
	Buono et al. [31]	90	No	No = 1	Yes	NR	2 days	NR	Yes	100 %	NR	Partial	NR
	Burns et al. [13]	134	Partial	Yes = 3	Yes	NR	NR	No	Yes	NR	NR	Partial	NR
	Castro-Piñeiro et al. [14]	2752	Partial	Yes = 18	NR	NR	NR	NR	Yes	95 %	NR	Yes	NR
	Cureton et al. [32]	753	Partial	NR	NR	NR	4 years	NR	Yes	99.30 %	NR	NR	NR
	Hunt et al. [33]	86	No	NR	Yes	NR	14 days	No	Yes	97 %	NR	Yes	Partial
	Mahar et al. [17]	266	Partial	Yes = 26	Yes	NR	7 days	Yes	No	NR	NR	Partial	NR

*NR* not reported

^a^Test battery

**Table 4:** Scalability of field based cardiovascular fitness tests

Assessment item	20-m shuttle run	1-mile walk/run
Delivery		
Ease of integration into usual service delivery	66.7 % (four teachers) had previous experience of test [34] Number of children that can be tested at once depends on space restrictions and capacity for timing individuals = 1 m width per child is recommended [35, 36]	Number of children that can be tested at once depends on space restrictions and capacity for timing individuals
Burden on delivery staff and other stakeholders	Considered feasible based on survey results from six PE teachers who were asked about factors relating to: (1) whether children wore appropriate clothing to perform, (2) ease of instructions, (3) ease of implementation, (4) rejections and appropriateness of facilities [34]	–
Preparation requirements	Two lines set up 20 m apart, speakers equal distance from each [36]	Measure distance if track unavailable
Test duration	Preparation = 5 min, testing = 10 min (a group of 20 individuals) [34]	Mean ± SD time for 8 = 11 years = 9.2 ± 1.8 mins (males), 10.3 ± 1.8 mins (females) [32]
Effectiveness		
Reach and adoption	Shown to be the preferable choice over the one mile run for student’s motivation for participation [37]. Students on average reported significantly higher situational interest in attention demand, exploration intention, and novelty in the 20-m shuttle run than one mile run [38]	Physical activity engagement (duration of activity, pace, energy expenditure) was significantly greater in the one mile run than the 20-m shuttle run, particularly for the low-performing students with a relatively high BMI [38]
Completion rates	One participant (*n* = 128) stopped due to lower body muscle cramp, tests were well tolerated, occurrence of severe DOMS in ten participants [34]	–
Resource requirements		
Equipment	Audio device, speakers, cones to mark length [36]	Stopwatch
Space	Flat surface, indoor (preferred) or outdoor (weather dependent), 20 m in length + room to turn round, 1-m width per child [36]	Outside measurable area, flat surface, no standard surface for this test therefore outdoor 400-m athletics track [10], dirt track [17], or grass athletics track [39] suitable
Human resource	Two members of staff = one to ensure protocols are followed correctly, one to record scores [36]	Two members of staff = one to time and one to record results [10, 17]
Training	CD provides audio instructions = no technical training required [36]	No advanced technical training requirements
Costs	–	–
Practical implementation issues and considerations	For a single study, 22 (37.9 %) children and 25 (33.3 %) adolescents experienced some degree of DOMS, from whom six children (10.3 %) and four adolescents (5.3 %) indicated that their DOMS was severe. Three (2.3 %) subjects reported having severe pain in the upper body, 29 (21.8 %) in the lower body and 14 (10.5 %) in the whole body. Most (39 participants; 29.3 %) assumed that the 20-m shuttle run test could be the cause. For 11 (19 %) children and 14 (18.7%) adolescents, DOMS caused difficulties in daily activities, especially stair climbing and walking [34]	Participants may have difficulty in developing an appropriate pace; participants may either start too fast so that they are not able to keep up the speed all through the test, or they may start too slow so that when they want to increase speed, the test is already finished [4]

*PE* physical education, *DOMS* delayed onset muscle soreness, *CD* compact disc, *SD* standard deviation, *BMI* body mass index

## Findings

A total of 229 studies reporting field-based tests of children’s cardiorespiratory fitness were identified. Initial analyses indicated that the 5-min run test did not meet the evidence criterion for reliability, whilst the 6-min walk test likewise failed to meet the evidence criterion for validity. A total of 25 studies remained for inclusion in the analysis. Of these, 19 reported the application of the 20-m shuttle-run, and six the one-mile walk/run (note that some studies considered more than one test) (Tables 2 and 3). A further four studies were identified that directly evaluated one or more aspects of scalability of field-based cardiorespiratory fitness tests for children and/or adolescents, and an additional five studies provided information on test protocols. These articles were used to complete the data extraction tables (Tables 3, 4).

Table 5 contains review items score totals for all included articles. For example, the table shows that out of the 25 articles, 8 % (*n* = 2) addressed practical implementation issues. A further 20 % (*n* = 5) received a partial score, with the reduction in rating predominantly due to the lack of information provided regarding practicality issues of administering the test, whilst 72 % (*n* = 18) reported no data relating to this variable.

**Table 5:** Assessment percentage scores for reviewed articles

Assessment item	Review items percentage score (%)			
Delivery	Yes	Partial	No	NR
Test context	72		16	12
Test duration	3			97
Testing interval	88			12
Delivery staff	20		68	12
Effectiveness				
Reach and adoption	85		15	
Completion rates	45			55
Cost considerations				
Cost effectiveness	0			100
Evidence of operating at scale				
Sample size	12	56	20	12
Number of schools	56		28	16
Resource requirements	11	65		24
Practical implementation issues/considerations	8	19		73

*NR* not reported

### Testing the Algorithm

**Fig. 2:**
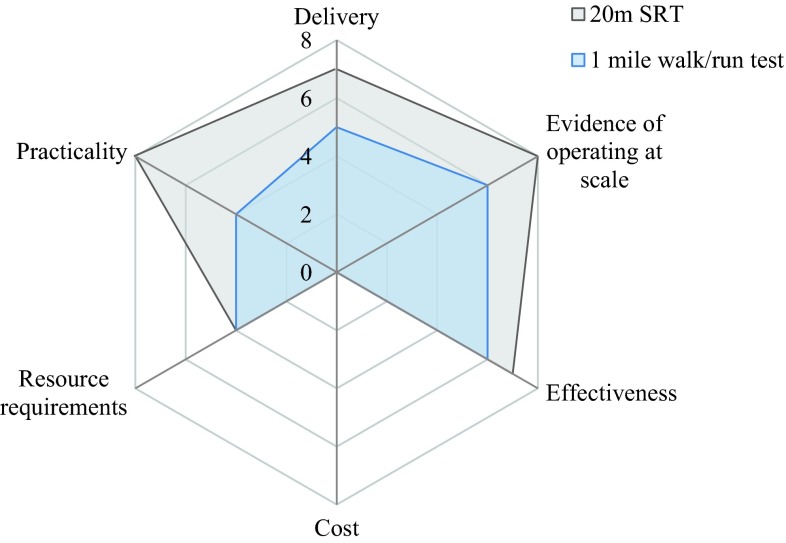
Scalability scores for 20m SRT compared with 1 mile walk/run test.* SRT* shuttle run test

The algorithm was used to rate the relative scalability of the 20-m shuttle run test and the 1-mile walk/run. Table 6 presents scores for each of the tests and Fig. 2 shows a spider diagram comparing component scores. The authors independently scored each test and a consensus meeting was arranged to interrogate and resolve any differences. The 20-m shuttle run test scored 34 of a possible 48 whilst the 1-mile walk/run scored 25. This indicates that of the two tests that met the criteria for validity and reliability, the 20-m shuttle run test is more scalable than the 1-mile walk/run test. However, a lack of information relating to cost-effectiveness/affordability of test delivery, economies of scale and marginal costs was evident and is discussed further below.

**Table 6:** Scalability scores for the 20-m shuttle run test and the 1-mile walk/run test

Component	Variable	20-m shuttle run test		1 mile run/walk	
		Score	Comment	Score	Comment
Delivery	Test context	2	14 studies conducted in school setting	2	Five studies conducted in school setting
	Test duration	1	One study reported = 90 mins (test battery)	0	Not reported
	Testing interval	2	19 studies used test for longitudinal testing, testing period range 7 days: 12 years	2	Four studies used test for longitudinal studies, range 7 days: 4 years
	Delivery staff	2	Three studies reported using PE staff to administer test	1	Two studies reported using PE staff to administer test
Total		7		5	
Evidence of operating at scale	Sample size	2	Three studies at population level (national, international), 13 studies multiple settings within local area	2	Three studies multiple settings within local area
	Number of schools	2	15 studies administered test in multiple schools (range 1–106)	1	Three studies administered test in multiple schools (range 1–26)
Total		4		3	
Effectiveness	Validity	2	Strong evidence [4]	1	Moderate evidence [4]
	Test–retest reliability	2	Strong evidence [5]	1	Moderate evidence [5]
	Reach and adoption	2	Reach and adoption across target groups and differential effect considered in 19 studies	2	Reach and adoption across target groups and differential effect considered in five studies
	Completion rates	1	Where reported completion rates varied from 74–100 %	2	Where reported completion rates varied from 97–100 %
Total score		7		6	
Cost	Cost-effectiveness	0	Not reported	0	Not reported
Total		0		0	
Resource requirements		1	Equipment = audio device, speakers, cones to mark length [36]. Space = flat surface, indoor (preferred) or outdoor (weather dependent), 20 m in length + room to turn round, 1-m width per child [36]. Human = 2 members of staff = one to ensure protocols are followed correctly, one to record scores [36]. Training = CD provides audio instructions = no technical training required [36]	1	Equipment = stopwatch. Space = outside measurable area, flat surface, no standard surface for this test therefore outdoor 400-m athletics track [10], dirt track [17], or grass athletics track [39] suitable. Human = two members of staff = one to time and one to record results [10, 17]. Training = no advanced technical training requirements
Total		1		1	
Practical implementation issues		2	For a single study, 22 (37.9 %) children and 25 (33.3 %) adolescents experienced some degree of DOMS, from which six children (10.3 %) and four adolescents (5.3 %) indicated that their DOMS was severe. Three (2.3 %) subjects reported having severe pain in the upper body, 29 (21.8 %) in lower body, and 14 (10.5 %) in the whole body. Most (39 participants; 29.3 %) assumed that the 20-m shuttle run test could be the cause. For 11 (19 %) children and 14 (18.7 %) adolescents, DOMS caused difficulties in daily activities, especially stair climbing and walking [34]	1	Participants may have difficulty in developing an appropriate pace; participants may either start too fast so that they are not able to keep up the speed all through the test, or they may start too slow so that when they want to increase speed, the test is already finished [4]
Total		2		1	
Overall weighted score		34		25	

*PE* physical education, *CD* compact disc, *DOMS* delayed onset muscle soreness

## Discussion

Year on year, greater emphasis is being placed on ensuring the real-world impact of scientific research, and the line between science and research on the one hand and policy and practice on the other is not as clearly defined as once it was. Scientists are increasingly expected to conduct research that not only reports traditional scientific metrics, but also data related to the real-world application of those, for example data pertaining to cost-effectiveness in health intervention research. A good example perhaps is that of Robertson et al. [40], who examined not only the validity and reliability of tests of skill in sport, important to those who use the data, but also the feasibility of the tests, equally important to those who conduct the testing.

Whilst the criteria of validity and reliability of children’s fitness tests are of major concern to scientists, the criterion of scalability is critical to policymakers and practitioners. As little is known about the scalability of fitness tests for children, in the present paper we presented data that will facilitate future decision making as to test provision, whilst also proposing a framework that could be applied to examine scalability in the context of either new data relating to existing tests or of data pertaining to new tests. Using this method we demonstrated that, based on available data, the 20-m shuttle run test is likely more scalable than the 1-mile walk/run test, with these tests scoring 34 and 25 of 48, respectively. However, a word of caution is required here given the stark contrast between the number of studies initially identified and the number of studies that met the inclusion criteria.

Whilst it is entirely understandable that scientific reports of fitness tests do not require the reporting of non-scientific data points such as costs, it is probably reasonable to suggest that with the increasing emphasis on real-world application and impact, it is incumbent on journal editors and reviewers, as well as policymakers and those funding research, to push for greater reporting of all such data where appropriate (this would perhaps be analogous to the way that the broader acceptance of meta-analysis as the gold standard of research synthesis has encouraged editors and funders to require the reporting effect sizes and/or all necessary data points to calculate these). We hope that this paper, by identifying the core components of scalability in the context of children’s fitness testing might encourage that process.

It is important to acknowledge limitations of the methodology reported. Firstly, as is the case with many if not most attempts at research synthesis, there was a stark contrast between the number of studies initially identified and the number of studies that met the inclusion criteria. This was likely compounded by our two-stage analysis. Without the reporting of all relevant data, however, it is problematic to evaluate scalability, and this was especially the case with regard to cost-effectiveness/affordability of test delivery, economies of scale, and marginal costs, for which no information could be found for either of the two fitness tests addressed in this study.

Second, in examining the literature we found only limited information on the definition of scalability and its core constituents. Therefore there are potentially one or more components of scalability that are not incorporated in our framework. For example, ethical consideration could be an important a priori factor in light of emerging web-based technologies.

Third, and related to the second, given this was a pioneering approach we had no a priori reason to justify weighting certain components within the framework more heavily than others. However it may be that in practice/application of the model, fundamental constraints to testing may evolve and the model may need to be developed accordingly. Such constraints may differ depending on who is applying the framework, for example whilst researchers may be more focused on ethics and controls, practitioners and policymakers may be more focused on costs.

## Conclusions

Recent systematic reviews by Castro-Piñero et al. [4] and Artero et al. [5] indicated strong support for the validity and reliability of the 20-m shuttle run test in the context of children’s fitness testing. Our analysis above should further encourage practitioners and policymakers to adopt this test either as an adjunct to, or replacement for, existing mandated tests such as the UK NCMP.

We also believe that the scalability framework developed in this paper has value beyond that of the context above. It has potential value in establishing the scalability of many types of fitness tests and/or measures, as well as in informing policy-makers in the up scaling of interventions from small projects or controlled trials to wider state, national or international programs.
